# Diversity, knowledge, and valuation of plants used as fermentation starters for traditional glutinous rice wine by Dong communities in Southeast Guizhou, China

**DOI:** 10.1186/s13002-019-0299-y

**Published:** 2019-04-27

**Authors:** Jianwu He, Ruifei Zhang, Qiyi Lei, Gongxi Chen, Kegang Li, Selena Ahmed, Chunlin Long

**Affiliations:** 1 College of Life and Environmental Sciences, Minzu University of China, Beijing, 100081 China; 2 Key Laboratory of Ethnomedicine (Minzu University of China), Ministry of Education, Beijing, 100081 China; 3 National and Local United Engineering Laboratory of Integrative Utilization Technology of Eucommia ulmoides, Jishou University, Jishou, 416000 Hunan China; 4 School of Health Science, Kaili University, Kaili, 556011 Guizhou China; 5 The Food and Health Lab, Department of Health and Human Development, Montana State University, Bozeman, MT 59717 USA; 6 Kunming Institute of Botany, Chinese Academy of Sciences, Kunming, 650201 China

**Keywords:** Ethnobotany, Traditional ecological knowledge, Rice wine, Fermented beverage, Fermentation starters

## Abstract

**Background:**

Beverages prepared by fermenting plants have a long history of use for medicinal, social, and ritualistic purposes around the world. Socio-linguistic groups throughout China have traditionally used plants as fermentation starters (or *koji*) for brewing traditional rice wine. The objective of this study was to evaluate traditional knowledge, diversity, and values regarding plants used as starters for brewing glutinous rice wine in the Dong communities in the Guizhou Province of China, an area of rich biological and cultural diversity.

**Methods:**

Semi-structured interviews were administered for collecting ethnobotanical data on plants used as starters for brewing glutinous rice wine in Dong communities. Field work was carried out in three communities in Guizhou Province from September 2017 to July 2018. A total of 217 informants were interviewed from the villages.

**Results:**

A total of 60 plant species were identified to be used as starters for brewing glutinous rice wine, belonging to 58 genera in 36 families. Asteraceae and Rosaceae are the most represented botanical families for use as a fermentation starter for rice wine with 6 species respectively, followed by Lamiaceae (4 species); Asparagaceae, Menispermaceae, and Polygonaceae (3 species respectively); and Lardizabalaceae, Leguminosae, Moraceae, Poaceae, and Rubiaceae (2 species, respectively). The other botanical families were represented by one species each. The species used for fermentation starters consist of herbs (60.0%), shrubs (23.3%), climbers (10.0%), and trees (6.7%). The parts used include the root (21.7%), leaf (20.0%), and the whole plant (16.7%). Findings indicate a significant relationship between knowledge of plants used as fermentation starters with age (*P* value < 0.001) and educational status (*P* value = 0.004) but not with gender (*P* value = 0.179) and occupation (*P* value = 0.059). The species that are most used by informants include *Pueraria lobata* var. *montana* (Lour.) van der Maesen (UV = 1.74; Leguminosae), *Actinidia eriantha* Benth. (UV = 1.51; Actinidiaceae), *Oryza sativa* L. var. *glutinosa* Matsum (UV = 1.5; Poaceae).

**Conclusion:**

This study highlights that while most of the Dong informants continue to use a diverse range of plants as a fermentation starter for brewing glutinous rice wine, knowledge of these plants is being lost by the younger generations. Documentation of traditional ethnobotanical knowledge and outreach is thus needed to conserve biocultural diversity in the rural Dong communities in southern China.

## Background

Fermented beverages have a long history of preparation and use globally for medicinal, social, and ritualistic purposes [1–4]. In China, different socio-linguistic groups in regions throughout the country have developed their own characteristic fermented beverages that are associated with cultural identity and social aspects of communities [3, 4]. For example, *Guyuelongshan* is a rice wine from Shaoxing in Zhejiang Province, *Hejiu* is a rice wine from Shanghai, and koumiss is a Mongolian liquor [5]. In addition, Tibetan communities prepare barley wine and there are many types of sweet rice wine from southwestern China including “*nuomi*” that are consumed during weddings, hospitality, funerals, ancestor worship, and other ceremonies [5].

Rice wine is among the most common and oldest fermented beverages in China. It is fermented using a fermentation starter, also known as *koji* (or *jiuqu* in Mandarin) [6]. *Koji* can be made with staple crops such as wheat, rice, millet, and maize that consist of microorganisms that support the fermentation process [7]. For example, communities in Shaoxing prepare *koji* as a raw material for rice wine from wheat that harbors many microorganisms including *Absidia*, *Acetobacteria*, *Aspergillus*, *Bacillus*, *Mucor*, *Lactobacillus*, and *Rhizopus* [8]. Some of these microorganisms are also used as single strains for the industrial manufacture of rice wine. Zhang et al. [9] highlighted that *Aspergillus oryzae* SU16, as a single strain, could be used in the production of *koji*.

In addition to common staple grains such as wheat, rice, millet, and maize for the preparation of fermentation starters, indigenous groups in mountainous regions of China have a long history of using a wide diversity of local plants for making *koji*. We previously documented a total of 103 species in 57 botanical families of wild plants that are traditionally used as starters for preparing fermented beverages by Shui communities in southwestern China [4]. The Dong are a socio-linguistic group (also known as the Kam) of southeast Guizhou that also have a long history of using *koji* for producing glutinous wine as a source of livelihood. Our previous studies demonstrate that the Dong people cultivate many varieties of glutinous rice [10, 11] which they use as their staple food. However, there remains a lack of documentation regarding the plants used as fermentation starters by Dong communities. This study seeks to address this knowledge gap by identifying the diversity of plants used as fermentation starters (*koji*) by Dong communities and associated knowledge and values. Findings have the potential to inform the conservation of natural resources associated with a culturally-relevant beverage of Dong communities while preserving traditional ecological knowledge.

## Methods

### Study area

**Fig. 1:**
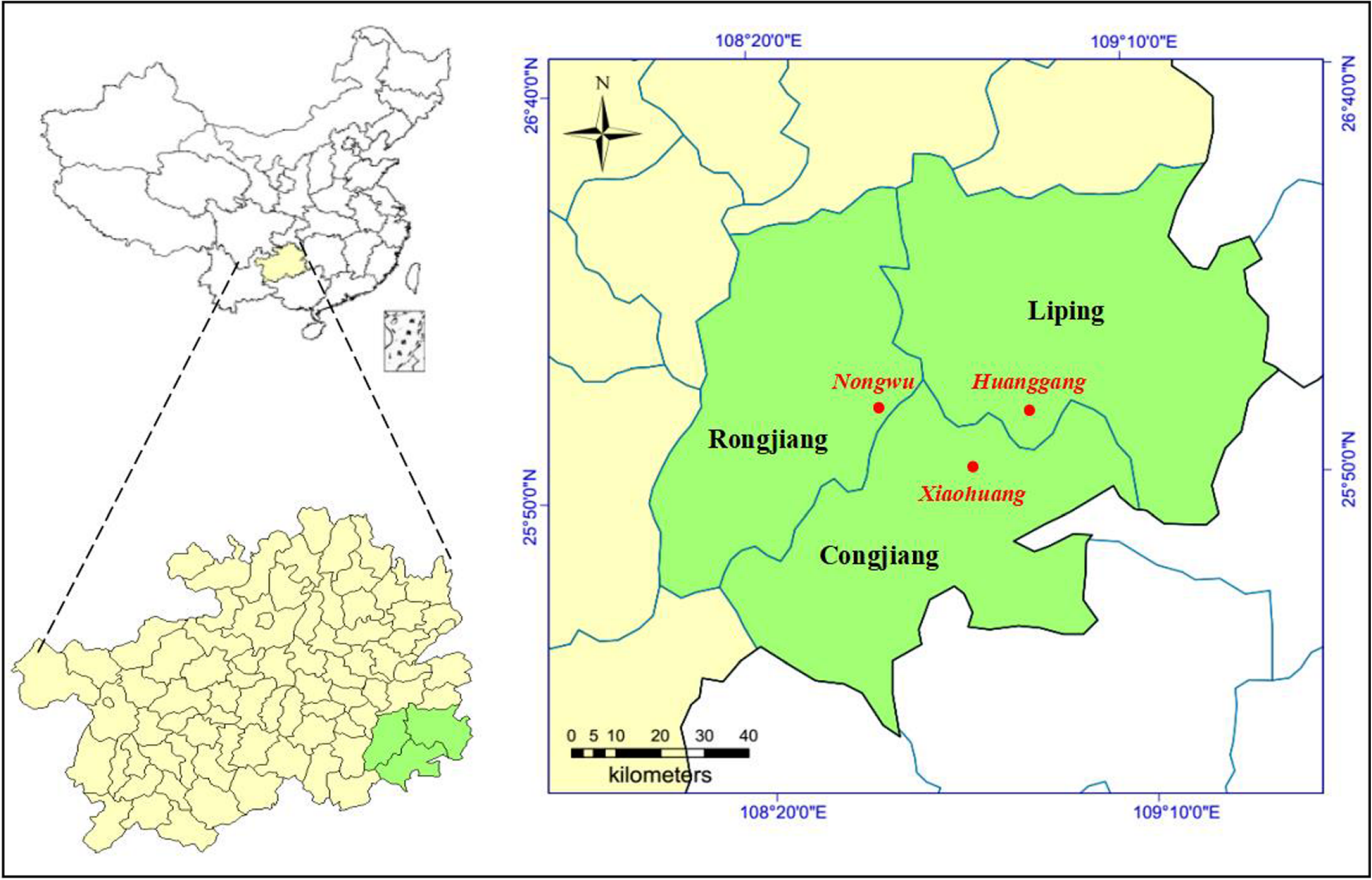
Geographic location of the study area: Xiaohuang, Huanggang, and Nongwu in three counties of Congjiang, Liping, and Rongjiang, respectively (Qiandongnan Miao and Dong Autonomous Prefecture, China)

Research was carried out in three Dong villages in Qiandongnan Miao and Dong Autonomous Prefecture in the southeastern part of Guizhou Province (longitude 108°50.3′ E–109°58.5′ E, latitude 25°53.7′ N–26°24.2′ N), located near Hunan and Guangxi provinces. These villages are in the core zone of Dong socio-linguistic group and include Xiaohuang of Congjiang County, Huanggang of Liping County, and Nongwu of Rongjiang County (Fig. 1, Table 1). The three villages have a combined area of 51.22 km^2^ and are located between 630 and 780 m above sea level. The climate is characterized as subtropical monsoon humid with an annual average temperature of 18.4 °C, an average precipitation of 1200 mm, average sunlight time of 1300 h, and a frost-free period of 310 days per year.

**Table 1:** Study area (three Dong villages in Qiandongnan Miao and Dong Autonomous Prefecture)

Village name	No. of family	Population	Area (km^2^)	Altitude (m)	Geographic location
Xiaohuang (Congjiang)	740	3800	16.53	630	25°53.7′ N, 109°58.5′ E
Huanggang (Liping)	325	1629	29.70	780	26°24.2′ N, 109°14.6′ E
Nongwu (Rongjiang)	135	550	4.99	740	25°94.1′ N, 108°50.3′ E

The three study site villages are dominated by members of the Dong and Miao socio-linguistic groups. Traditional rice-fish co-culture system predominates in these villages and integrates with animal husbandry, forestry management, and medicinal plant collection and trade [10, 11]. In this study, we selected to focus on interviewing Dong households because of their longer history of cultivating glutinous rice (*Oryza sativa* var. *glutinosa*) compared to the Miao as well as their subsistence lifestyle for procuring food. Glutinous rice wine is a very popular fermented beverage in local communities. The Dong, as many indigenous communities, rely on their environment for a range of wild and cultivated crops for preparing food, beverages, and medicine [12, 13]. The above information indicates that these villages are ideal areas for studying the traditional knowledge of plants used as fermentation starters for traditional glutinous rice wine.

### Ethnobotanical data collection

Ethnobotanical surveys were carried out from September 2017 to July 2018. A total of 217 informants (including 126 male and 91 female) were interviewed from the three study sites (Table 2). Semi-structured interviews were carried out using a snowballing approach of meeting Dong community members including in fields, around fish ponds, in canteens, in artisanal workshops, in farmhouses, and in village squares. The semi-structured interviews involved open-ended questions and conversations with informants in the above scenes. The major questions are as follows:

1. Do you know about “*Jiuqu*” (fermentation starters for brewing traditional glutinous rice wine)?
2. Do you know the technology of koji-making?
3. If yes, which plants did you choose, and which parts of the plants to make the fermentation starters?
4. Where do you usually collect these plants?
5. Can you take us to collect these plants? (Field identification or local plant flora).
6. Do you know these plant names?
7. Can you read these names in Dong language?
8. Why do not you choose a commercial “*Jiuqu*” for brewing traditional glutinous rice wine?
9. Would you consider passing this knowledge to your children or other people?
10. What other interesting things can you share with us?

**Table 2:** Demographic details of interviewed informants

Category	Subcategory	Number of informants	% of informants
Gender	Male	126	58.06
	Female	91	41.94
Age	20–40	22	10.14
	40–60	117	53.92
	60 and older	78	35.94
Education status	Illiterate	152	70.05
	Primary	44	20.28
	Secondary	16	7.37
	Higher	5	2.30
Occupation	Farmer	133	61.29
	Migrant workers	71	32.72
	Local officials	13	5.99
Knowledge about koji-making plants	Yes	193	88.94
	No	24	11.06

**Fig. 2:**
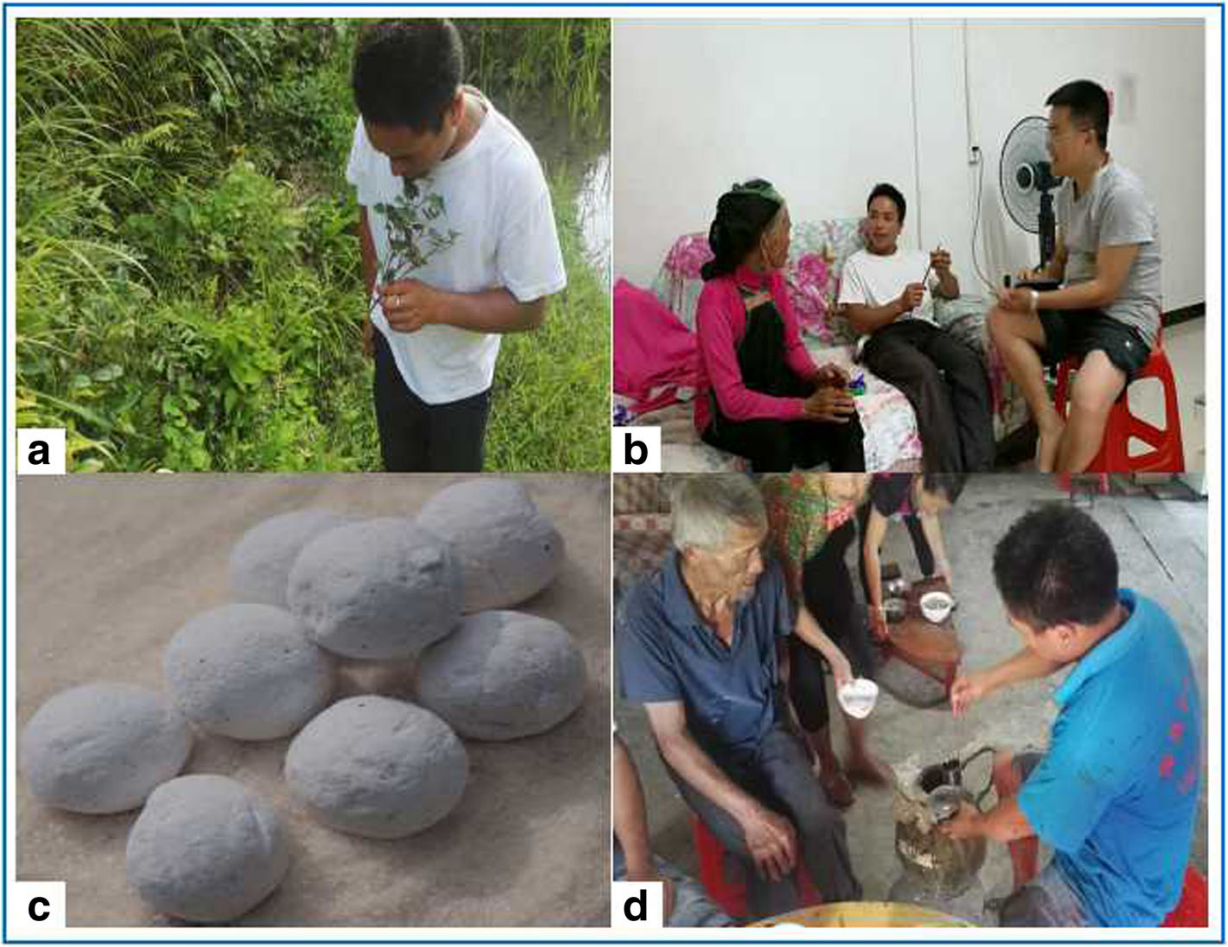
Indigenous knowledge of traditional glutinous rice wine koji-making plants: **a** A local guide to helping identification of glutinous rice wine koji-making plants. **b** One of face-to-face interview. **c** The koji for brewing glutinous rice liquor/wine. **d** Glutinous rice wine made from koji

Interviews were carried out in either the Dong language with the assistance of a local Dong translator (Fig. 2) or in simplified Mandarin.

In the local area, people with primary and higher education tend to go out to work as migrant workers in non-agriculture times, and those with higher education have the opportunity to find permanent jobs in the provincial and prefectural capital cities, or county towns nearby. Interviews in Mandarin were primarily with individuals with primary education or above including migrant workers and local government officials. All interview procedures involved in this study were in accordance with the International Society of Ethnobiology Code of Ethics including procuring prior informed consent before interviews [14]. The demographic characteristics (age, educational status, and occupation) were identified and recorded in all face-to-face interviews (Fig. 2).

In addition to interviews, we carried out participatory observation in the study site communities. Specifically, we focused on observing the process of collecting plants and preparing *koji*. These observations were supplemented by key informant interviews on the type of plant species. All of the plants mentioned by key informants were identified in the field and collected to prepare voucher specimens. We checked the scientific names of our field collections with *The Plant List* [15]. Botanical specimens were further examined at the Herbarium of Jishou University, Hunan Province, China. The specimens were assigned voucher numbers and deposited at the Herbarium of Jishou University.

### Data analysis

Classical ethnobotanical descriptive statistics were used to summarize ethnobotanical data in Excel 2013. The association between indigenous knowledge of koji-making with participant’s demographic factors including gender, age, educational status, and occupation was tested with Chi-square analysis. Statistical analysis was carried out using SPSS version 20 (SPSS, Chicago) at 5% level of significance (*P* < 0.05). Use Value (UV) index [16] was calculated to evaluate the botanical species with the greatest use across the study site communities. The UV of each plant mentioned was calculated using the following formula:

$$ \mathrm{UV}=\frac{\sum \mathrm{UP}}{\ n} $$

where UP is the number of uses mentioned by each informant for a given plant use and *n* is the total number of informants.

## Results

### Socio-demographic characteristics of respondents

Table 2 describes the demographic characteristics of the 217 study informants. Informants comprised of 58.06% (*N* = 126) males and 41.94% (*N* = 91) females. In addition, informants were between the ages of 20 and 96 years (the majority were between 40 and 60 years old). Most of the surveyed respondents (70.05%) are illiterate, and only five (2.30%) of the interviewed respondents had completed higher education (Table 2). The majority of the respondents were farmers (61.29%, *N* = 133) and migrant workers (32.72%, *N* = 71), except for a few local government officials (5.99%, *N* = 13). Most respondents (*N* = 193; 88.94%) demonstrated average knowledge about *koji* plants in general (Tables 2 and 3).

**Table 3:** Knowledge about koji-making plants in relation with gender, age, educational status, and occupation of the respondents

Characteristics	Total number of respondents	Knowledge about koji-making plants		*X* ^2^	*P* value
		Yes	No		
Gender				*X*^2^ = 1.807, df = 1	*P* = 0.179
Male	126	109	17		
Female	91	84	7		
Age				*X*^2^ = 58.668, df = 2	*P <* 0.001
20–40	22	9	13		
40–60	117	108	9		
60 and older	78	76	2		
Education status				*X*^2^ = 13.443, df = 3	*P* = 0.004
None	152	141	11		
Primary	44	38	6		
Secondary	16	11	5		
Higher	5	3	2		
Occupation				*X*^2^ = 5.664, df = 2	*P* = 0.059
Farmers	133	119	14		
Migrant workers	71	65	6		
Local officials	13	9	4		

### Diversity of plants used for *koji*

A total of 60 plant species were documented for preparing *koji*, belonging to 58 genera and 36 families (Table 4). The most prevalent botanical families were Asteraceae and Rosaceae (*N* = 6, respectively), followed by Lamiaceae (*N* = 4); Asparagaceae, Menispermaceae, and Polygonaceae (*N* = 3, respectively); Lardizabalaceae, Leguminosae, Moraceae, Poaceae, and Rubiaceae (*N* = 2, respectively); and the other botanical families represented in our collections each consisted of a single species (Table 4).

**Table 4:** Inventory of plants traditionally used for koji-making in the study area (species are listed alphabetically)

Scientific name	Voucher number	Family name	Dong name	Chinese name	Habit	Part used	UV
*Actinidia eriantha* Benth.	KJBT0040	Actinidiaceae	Sangp buc dongl	Mao Hua Mi Hou Tao	Shrub	Branch	1.51
*Adiantum flabellulatum* L.	KJBT0052	Pteridaceae	Kaok naeml	Shan Ye Tie Xian Jue	Herb	Leaf	0.79
*Agrimonia pilosa* Ledeb	KJBT0029	Rosaceae	Demh Meix Sais	Lu Bian Huang	Herb	Root	0.47
*Akebia quinata* (Houtt.) Decne.	KJBT0064	Lardizabalaceae	Gueel nyanl bads	Ba Yue Gua	Shrub	Fruit	0.67
*Arctium lappa* L.	KJBT0027	Asteraceae	Mal kap gueec	Niu Bang	Herb	Aerial part	0.45
*Artemisia annua* L.	KJBT0019	Asteraceae	Mal yaems sul	He Hao	Herb	Root	1.19
*Asarum forbesii* Maxim.	KJBT0033	Aristolochiaceae	Naos max tic	Ma Ti Xiang	Herb	Leaf	0.77
*Asparagus cochinchinensis* (Lour.) Merr.	KJBT0050	Asparagaceae	Sangp begs sangp laox	Tian Men Dong	Herb	Root	0.56
*Bauhinia brachycarpa* Wall. ex Benth.	KJBT0059	Leguminosae	Jaol bav	Ye Guan Men	Shrub	Root	0.47
*Cayratia trifolia* (L.) Domin	KJBT0023	Vitaceae	Jaol meixguv	San Ye Wu Lian Mei	Shrub	Fruit	1.25
*Cirsium japonicum* DC.	KJBT0044	Asteraceae	Mal sax bav laox	Da Ji	Herb	Root	0.31
*Clerodendrum cyrtophyllum* Turcz.	KJBT0009	Lamiaceae	Bav sup geel kuenp	Da Qing Ye	Shrub	Aerial part	0.44
*Codonopsis pilosula*	KJBT0011	Campanulaceae	Demh Gaams Yous	Dang Shen	Climber	Root	0.40
*Cunninghamia lanceolata* (Lamb.) Hook.	KJBT0047	Taxodiaceae	Meix beens	Sha Mu Ye	Tree	Leaf	1.24
*Cyclea racemosa* Oliv.	KJBT0002	Menispermaceae	Jaol enl sup dangl	Lun Huan Teng	Herb	Branch	0.79
*Diospyros cathayensis* Steward	KJBT0048	Ebenaceae	Meix bav minc	Shi Zi Ye	Tree	Leaf	0.86
*Elaeagnus pungens* Thunb.	KJBT0051	Elaeagnaceae	Demh nyox senc	Hu Tui Zi	Shrub	Aerial part	0.78
*Fallopia multiflora* (Thunb.) Harald.	KJBT0018	Polygonaceae	Jaol maenc yeex	He Shou Wu	Climber	Root	0.28
*Ficus pumila* L.	KJBT0006	Moraceae	Jaol liangc fenx	Cheng Tuo Guo	Tree	Leaf	0.27
*Ficus tikoua* Bur.	KJBT0013	Moraceae	Jaol demh xeens	Di Gua Teng	Climber	Whole plant	0.47
*Gardenia jasminoides* Ellis	KJBT0022	Rubiaceae	Wap lagx ngoc	Huang Zhi Zi	Shrub	Flower	1.03
*Gaultheria leucocarpa* Bl. var. *crenulata* (Kurz) T. Z. Hsu	KJBT0053	Ericaceae	Melx demh miuus	Bai Zhu Shu	Herb	Leaf	1.25
*Gentiana rhodantha* Franch. ex Hemsl.	KJBT0028	Gentianaceae	Nyangt boy liongc	Long Dan Cao	Herb	Whole plant	1.21
*Gerbera piloselloides* (L.) Cass.	KJBT0034	Asteraceae	Sangp mal kap gav	Mao Da Ding Cao	Herb	Whole plant	1.00
*Geum macrophyllum* Willd.	KJBT0030	Rosaceae	Yangh muic naemx	Lu Bian Qing	Herb	Aerial part	0.92
*Glochidion puberum* (Linn.) Hutch.	KJBT0049	Phyllanthaceae	Meix sonp ponc	Suan Pan Zi	Tree	Fruit	1.07
*Gonostegia hirta* (Bl.) Miq.	KJBT0038	Urticaceae	Mal kgoux lail	Nuo Mi Tuan	Herb	Whole plant	0.92
*Hedera nepalensis* var. *sinensis* (Tobl.) Rehd.	KJBT0005	Araliaceae	Jaol bav yaop	Chang Chun Teng	Shrub	Aerial part	0.40
*Houttuynia cordata* Thunb.	KJBT0063	Saururaceae	Sangp wadc	Zhe Er Gen	Herb	Root	1.46
*Imperata cylindrica* (L.) Beauv.	KJBT0003	Poaceae	Sangp nyangt bagx	Bai Mao Gen	Herb	Root	1.12
*Kadsura longipedunculata* Finet et Gagnep.	KJBT0046	Schisandraceae	Jaol dangl bogl padt	Shan Wu Wei Zi	Shrub	Bark	1.47
*Kalimeris indica* (L.) Sch.-Bip.	KJBT0032	Asteraceae	Mal langx	Ni Qiu Chuan	Herb	Aerial part	0.76
*Leonurus japonicus* Houtt.	KJBT0060	Lamiaceae	Mal semp beengc	Yi Mu Cao	Herb	Whole plant	0.96
*Ligularia fischeri* (Ledeb.) Turcz.	KJBT0042	Asteraceae	Bav dinl max	Ti Ye Tuo Wu	Herb	Branch	0.46
*Melastoma dodecandrum* Lour.	KJBT0014	Melastomataceae	Mal demh xeens	Di Shen	Shrub	Leaf	0.79
*Mentha canadensis*	KJBT0043	Lamiaceae	Naos suic yeex	Bo He	Herb	Leaf	1.46
*Oryza sativa* var. *glutinosa* Matsum.	KJBT0037	Poaceae	Oux	Nuo He	Herb	Stem	1.50
*Paris polyphylla* Smith	KJBT0039	Melanthiaceae	Wap bar Yeal	Qi Ye Yi Zhi Hua	Herb	Whole plant	0.55
*Polygala sibirica* L.	KJBT0017	Polygalaceae	Sangp jeml meec angh	Gua Zi Jin	Herb	Aerial part	0.82
*Polygonatum cyrtonema* Hua	KJBT0021	Asparagaceae	Xingp mant jenc	Huang Jing	Herb	Root	1.00
*Polygonum hydropiper* L.	KJBT0026	Polygonaceae	Meix bav	La Liao	Herb	Leaf	1.42
*Portulaca oleracea* L.	KJBT0016	Portulacaceae	Mal Nguedc	Gua Zi Cai	Herb	Whole plant	1.00
*Pteridium aquilinum* (L.) Kuhn var. *latiusculum* (Desv.) Underw. ex Heller	KJBT0062	Dennstaedtiaceae	Kaok	Jue Cai	Herb	Stem	0.92
*Pueraria lobata* var. *montana* (Lour.) van der Maesen	KJBT0015	Leguminosae	Sangp nieengv	Ge Teng	Climber	Branch	1.74
*Frangula crenata* (Siebold & Zucc.) Miq.	KJBT0024	Rhamnaceae	Meix liuucliic	Ku Li Ye	Shrub	Leaf	1.22
*Rohdea japonica* (Thunb.) Roth	KJBT0054	Asparagaceae	Mal nyinc sup	Wan Nian Qing	Herb	Root	1.12
*Rosa laevigata* Michx	KJBT0065	Rosaceae	Ongv kuaot	Jin Ying Zi	Shrub	Fruit	1.38
*Rosa roxburghii* Tratt.	KJBT0007	Rosaceae	Sunl ongv kuaot	Ci Li	Herb	Fruit	1.44
*Rubus pluribracteatus* L.T.Lu & Boufford.	KJBT0008	Rosaceae	Demh bav daemh gal	Da Hei Mei	Climber	Fruit	1.42
*Sanguisorba officinalis* L.	KJBT0020	Rosaceae	Sangp lagx lugx yak	Hong Di Yu	Herb	Root	0.81
*Sargentodoxa cuneata* (Oliv.) Rehd. et Wils.	KJBT0057	Lardizabalaceae	Jaol bogl padt yak mags	Xue Teng	Herb	Branch	1.20
*Solanum americanum* Mill.	KJBT0025	Solanaceae	Lianh yeex	Ye Hai Jiao	Herb	Fruit	1.29
*Stephania cepharantha* Hay.	KJBT0045	Menispermaceae	Sunl maenc jinc	Jin Xian Diao Wu Gui	Herb	Root	0.88
*Teucrium quadrifarium* Buch.-Ham. ex D. Don	KJBT0036	Lamiaceae	Nyangt ous	Niu Wei Cao	Herb	Whole plant	0.46
*Thalictrum microgynum* Lecoy. ex Oliv.	KJBT0056	Ranunculaceae	Wangc lieenc naemx	Xiao Guo Tang Song Cao	Herb	Whole plant	0.45
*Tinospora sagittata Gagnep*.	KJBT0004	Menispermaceae	Sangp juc saengc	Qing Niu Dan	Shrub	Leaf	0.47
*Uncaria rhynchophylla* (Miq.) Miq. ex Havil.	KJBT0010	Rubiaceae	Sangp jaol kgoul daov	Da Ye Gou Teng Ye	Climber	Branch	1.32
*Verbena officinalis* L.	KJBT0031	Verbenaceae	Nyangt piudt max bieenh	Ma Bian Cao	Herb	Leaf	0.79
*Viola philippica* Cav.	KJBT0012	Violaceae	Mal mac keip	Di Cao Guo	Herb	Whole plant	0.47
*Zanthoxylum bungeanum* Maxim.	KJBT0066	Rutaceae	Sangp siul yanl	Hua Jiao	Shrub	Fruit	0.92

**Fig. 3:**
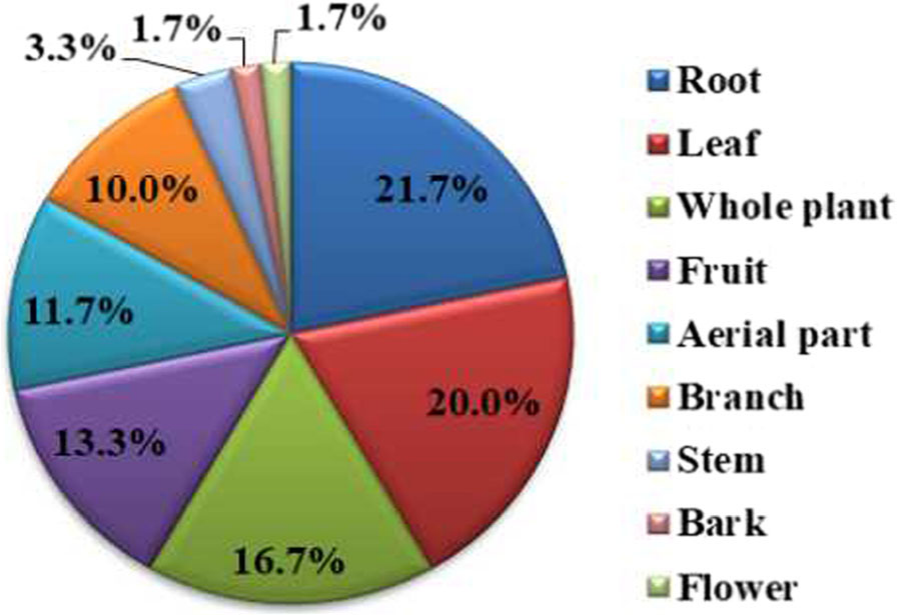
Percentage of koji-making plant parts used

Analysis of the life forms of koji-making plants showed that 60.0% of the reported species are herbaceous plants (*N* = 36), 23.3% are shrubs (*N* = 14), 10.0% are lianas (10.0%), and 6.7% are trees (*N* = 4) (Table 4). The root was the most commonly used plant part (21.7%, *N* = 13 citations), followed by the leaf (20.0%, *N* = 12), whole plant (16.7%, *N* = 10), fruit (13.3%, *N* = 8), aerial part (11.7%, *N =* 7), branch (10.0%, *N =* 6), stem (3.3%, *N* = 2), bark, and flower (1.7%, *N* = 1, both) (Table 4, Fig. 3).

### Traditional knowledge on koji-making plants

Results of the Chi-square test showed that there was no significant association between knowledge of the koji-making plants and gender (*X*^2^ = 1.807, df = 1, *P* value = 0.179) and occupation (*X*^2^ = 5.664, df = 2, *P* value = 0.059). However, there was a significant association between knowledge of *koji* plants with age (*X*^2^ = 58.668, df = 2, *P* value < 0.001) and educational status (*X*^2^ = 13.443, df = 3, *P* value = 0.004) (Table 3). Informants older than 40 years and those with lower educational status were the most knowledgeable regarding plants for making *koji* (Table 3).

### Frequently utilized species

The use values (UV) calculated for this study range from 0.27 to 1.74, with a higher UV indicating the plant was more frequently reported to be used by informants. The plant species most frequently utilized by informants for making *koji* are *Pueraria lobata* var. *montana* (Lour.) van der Maesen (1.74), *Actinidia eriantha* Benth. (1.51), and *Oryza sativa* L. var. *glutinosa* Matsum (1.5). There were 23 other species with a UV value greater than 1 including *Kadsura longipedunculata* Finet et Gagnep, *Houttuynia cordata* Thunb., *Mentha canadensis* L., *Rosa roxburghii* Tratt, *Polygonum pubescens* (Meissn.) Steward, *Rubus pluribracteatus* L.T. Lu & Boufford, *Rosa laevigata* Michx, *Uncaria rhynchophylla* (Miq.) Miq. ex Havil, *Solanum americanum* Mill., *Cayratia trifolia* (L.) Domin, *Gaultheria leucocarpa* Bl. var. *crenulata* (Kurz) T. Z. Hsu, *Cunninghamia lanceolata* (Lamb.) Hook, *Frangula crenata* (Siebold & Zucc.) Miq., *Gentiana rhodantha* Franch. ex Hemsl, *Sargentodoxa cuneata* (Oliv.) Rehd. et Wils, *Artemisia annua* L., *Imperata cylindrica* (L.) Beauv, *Rohdea japonica* (Thunb.) Roth, *Glochidion puberum* (L.) Hutch, *Gardenia jasminoides* Ellis, *Gerbera piloselloides* (L.) Cass, *Polygonatum cyrtonema* Hua, and *Portulaca oleracea* (L.) (Table 4).

## Discussion

The technique of using plants as fermentation starters is a prevalent traditional method for preparing many well-known fermented foods and beverages in China [17, 18]. This study highlights the diversity of plants used by Dong communities as fermentation starters for making rice wine as well as associated knowledge and use value based on the most frequently reported plants used for *koji*. We documented a total of 60 plant species and associated plant parts used by informants in the Dong study site communities as fermentation starters for making glutinous rice wine. Our results further showed that 88.94% of respondents had knowledge about plants used as fermentation starters. This finding indicates the rich indigenous ecological knowledge regarding plants in Dong communities which contributes to sustaining livelihoods and well-being along with biodiversity.

Many informants claimed “People who cannot make glutinous rice wine are not a real Dong people, because drinking and singing become a part of our daily life.” This naive view clearly emphasized the importance of fermented beverages in Dong communities and partially suggested that the koji for brewing *glutinous rice wine* was widely used in the area. Our results further showed that there was no significant difference in knowledge of *koji* plants between gender or social occupation. These results suggest that *koji* plants are generally known by local people irrespective of their gender or job.

An older informant (the old woman in red shirt in Fig. 2b) said “Glutinous rice wine is easy to brew, but making koji is a profound knowledge that young people won’t understand.” This statement has been cross-validated among several other informants. Interestingly, the results of this survey showed a significant association between knowledge of *koji* plants and the respondent’s age, indicating that elder people have more knowledge about *koji* plants than young people.

Although our results showed there was a significant negative correlation between the education level of respondents and the traditional knowledge on *koji* plants they possess, findings from this study are in line with another study that shows that educational status does not contribute to the mastery of traditional ecological knowledge [19]. But we cannot conclude that the education status decreased this traditional knowledge. Because the ratio of educated informants was too small, while education is more or less related to age (the younger people are more educated than older ones). It is worth mentioning that, in the study area, many young community members intend to go to distant cities for higher education from an early age. Thus, their communication with elders about traditional glutinous rice wine *koji* plants is limited.

The 60 species documented in this study represent a diverse range of botanical genera; specifically, the *koji* plants belong to 58 genera and 36 families with the dominant families including Asteraceae, Rosaceae, Lamiaceae, Asparagaceae, Menispermaceae, and Polygonaceae. A comparison of findings from this study with other regional surveys on plants used as fermentation highlights how species composition and diversity notably varies on the basis of cultural group. A survey by Hong et al. [4] with the Shui socio-linguistic group, also in Guizhou Province, documented that respondents harvested 103 wild plant species in 88 genera and 57 families used as starters for preparing fermented beverages. The majority of plants belonged to the families Asteraceae, Rosaceae, Fabaceae, Melastomaceae, Moraceae, and Rutaceae. For example, Shui communities have been shown to use 9 species in the Rosaceae as fermentation starters (*Agrimonia pilosa* Ledeb., *Geum aleppicum* Jacq., *Rosa roxburghii* Tratt., *Rosa laevigata* Michx., *Rubus alceaefolius* Poir., *Rubus corchorifolius* L., *Rubus ellipticus* Sm., *Rubus xanthocarpus* Bureau & Franch., and *Rubus niveus* Thunb.) while Dong communities use 6 species in the family for *koji* (*Agrimonia pilosa* Ledeb., *Geum macrophyllum*, *Rosa laevigata* Michx., *Rosa roxburghii* Tratt., *Rubus pluribracteatus* L., and *Sanguisorba officinalis* L.). This comparison demonstrates the distinctiveness in species composition among different socio-linguistic groups within the same region (Guizhou Province) of China. Through our interviews, we got a general understanding of traditional technology of local starter-making. They roughly mashed the cleaned plants and plant parts with a wooden hammer, then stirred the powder of the glutinous rice shell into the mixture until mixing, and then rubbed or rolled the mixture into a bolus between hands. After wetting the surface of the bolus with water from mountain springs, they put the mixture in a barrel and let it ferment naturally, and then place it in indoors for air drying after the surface of bolus has grown white mold.

At the same time, a comparison of findings from this study with other regional surveys on plants highlights how species composition and diversity may also show convergence between cultural groups. Specifically, the species composition found in this study has notable congruence to the general floristic profile of Miao community reported by Liu et al., which revealed that the Rosaceae, Asteraceae, Poaceae, and Liliaceae were dominant botanical families in Puding, Guizhou Province [20]. The analysis of the community structure of local plants in the study area confirms the rationality of the versatility hypothesis of Gaoue et al. [21]. The traditional practice of plant uses, along with the enhancement of the brewing technology, contributes to the diversity and complexity in the use of *koji* plants by the Dong.

As species and family level alone are not enough to comprehensively understand the keystone ethnobotanical species of *koji* plants, a quantitative evaluation method of calculating use values (UV) was applied in this study. UV is a commonly used indicator in the fields of ethnobotany and ethnoecology [15]. The evaluation of UV has the potential to reveal the utilization value of plant species and identify culturally-important plant resources [18]. Findings on UV in Dong communities showed that some parts of plant species had very restricted uses. For example, stems of *Pueraria lobata* var. *montana*, *Actinidia eriantha*, and glutinous rice were not reported in any published ethnobotanical studies as food or food raw materials. Alternatively, we found some *koji* plants widely reported in the literature as edible wild vegetables or fruits while having limited commercial use in the study area. Examples of these plants include *Artemisia annua* [22], *Elaeagnus pungens* [23], *Houttuynia cordata* [24], *Portulaca oleracea* [25], *Pteridium aquilinum var*. *latiusculum* (bracken fern) [26], *Rosa laevigata*, and *Rosa roxburghii* [27]. Additionally, we identified multiple other plants used by study informants for *koji* that have not been reported for this use in other geographical and sociocultural contexts, including “Naos suic yeex” (*Mentha canadensis*) and “Sangp siul yanl” (*Zanthoxylum bungeanum*). *Mentha canadensis* is a widely used plant to extract essential oil [28] and is also consumed in China for medicinal purposes in treating human diseases and to enhance appetite. The fruit of *Zanthoxylum bungeanum* is popular as a seasoning and traditional Chinese herbal medicine, and widely distributed in China and some Southeast Asian countries [29].

## Conclusion

This study highlights that the majority of Dong informants in the study site communities continue to use a wide diversity of plants as fermentation starters for brewing glutinous rice wine, a tradition that is over a thousand years old. In addition, this study highlights that elders in study site communities continue to have richer traditional ecological knowledge regarding plants used as fermentation starters and that this knowledge is not being transmitted to the younger generation. The most prevalent *koji* plants reported in this study include *Pueraria lobata* var. *montana* (Lour.) van der Maesen stem, *Actinidia eriantha* Benth., and *Oryza sativa* var. *glutinosa* Matsum. stem. Findings of this study can be used to inform programs focused on the preservation of botanical resources used for preparing traditional glutinous rice wine edge. Similar to our findings of dye plants in the Dong area [30], we suggest supporting educational workshops and training focused on transmitting the traditional ecological knowledge of community elders to the younger generation. It is expected that such efforts will not only support the cultural identity of communities through the preservation of knowledge and practices, but will also help conserve surrounding biodiversity that is embedded in traditional ecological knowledge.
